# Supplementary material for: Predicting visual function by interpreting a neuronal wiring diagram
Source: Nature. 2024 Oct 2;634(8032):113–23. doi: 10.1038/s41586-024-07953-5 (PMC11446822; doi:10.1038/s41586-024-07953-5)
Supplement: Supplementary file 6 — CRF and ERF predictions for individual TmY4 and TmY9 cells. Analogous to Supplementary Data 3, but for TmY target types. Shown are the top four monosynaptic pathways, the strongest pathway passing through each of the top ten intermediary types (ranking from Extended Data Fig. 7), and the trisynaptic pathway Tm1–TmY–Dm3–TmY (see the section entitled Prediction of spatial normalization). [file 41586_2024_7953_MOESM6_ESM.zip › DataS4/TmY9qΓèÑ/720575940628225256.pdf]

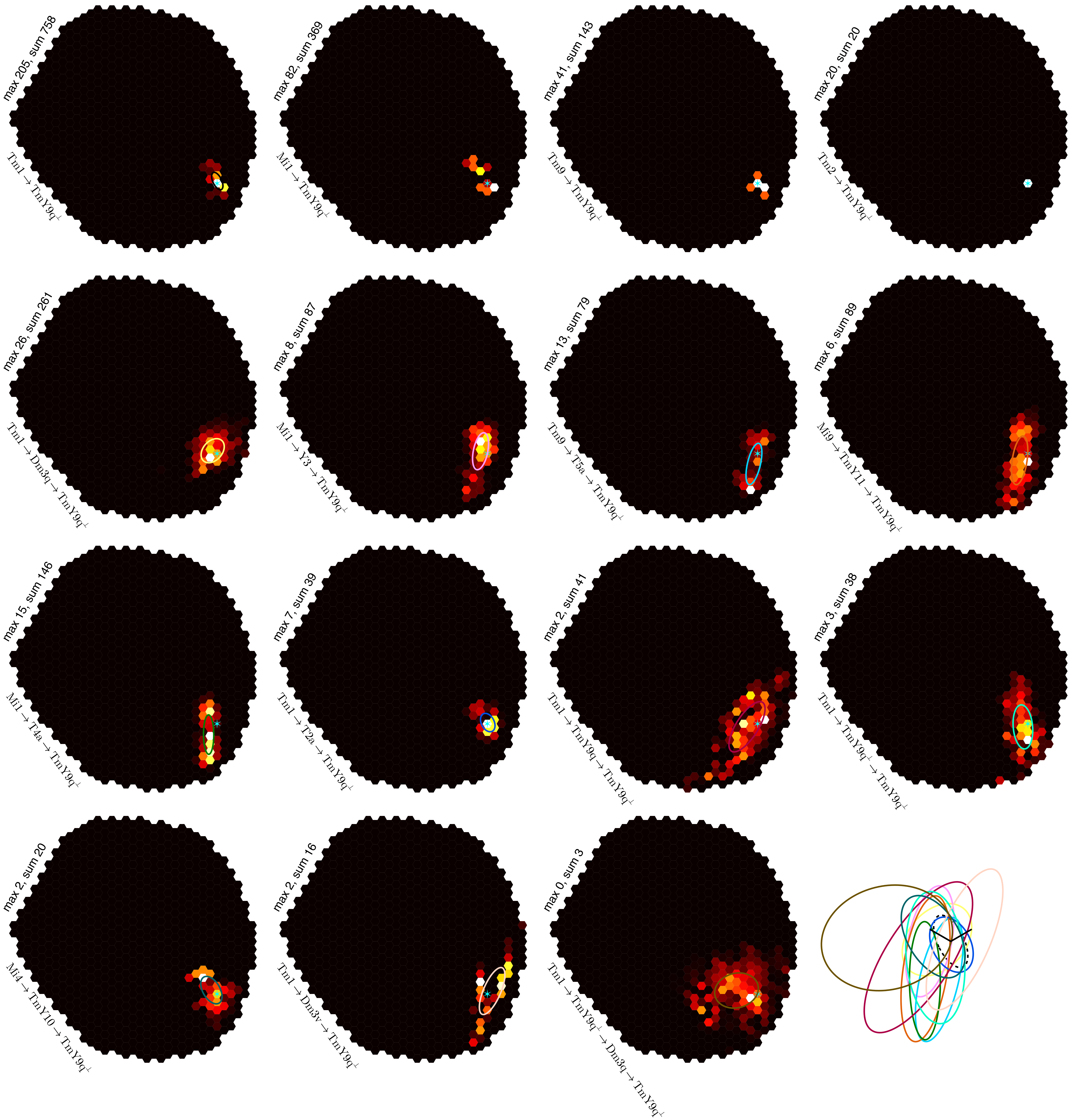

max 205, sum 758
Tm1 → TmY9q⊥
max 82, sum 369
Mi1 → TmY9q⊥
max 41, sum 143
Tm9 → TmY9q⊥
max 20, sum 20
Tm2 → TmY9q⊥
max 26, sum 261
Tm1 → Dm3q → TmY9q⊥
max 8, sum 87
Mi1 → Y3 → TmY9q⊥
max 13, sum 79
Tm9 → T5a → TmY9q⊥
max 6, sum 89
Mi9 → TmY11 → TmY9q⊥
max 15, sum 146
Mi1 → T4a → TmY9q⊥
max 7, sum 39
Tm1 → T2a → TmY9q⊥
max 2, sum 41
Tm1 → TmY9q → TmY9q⊥
max 3, sum 38
Tm1 → TmY9q⊥ → TmY9q⊥
max 2, sum 20
Mi4 → TmY10 → TmY9q⊥
max 2, sum 16
Tm1 → Dm3v → TmY9q⊥
max 0, sum 3
Tm1 → TmY9q⊥ → Dm3q → TmY9q⊥
